# Association study of SNP locus for color related traits in herbaceous peony (*Paeonia lactiflora Pall.*) using SLAF-seq

**DOI:** 10.3389/fpls.2022.1032449

**Published:** 2022-12-05

**Authors:** Genzhong Liu, Ying Li, Xia Sun, Xianfeng Guo, Nannan Jiang, Yifu Fang, Junqiang Chen, Zhilong Bao, Fangfang Ma

**Affiliations:** ^1^ State Key Laboratory of Crop Biology, College of Horticulture Science and Engineering, Shandong Agricultural University, Tai-An, Shandong, China; ^2^ College of Forestry, Shandong Agricultural University, Tai-An, Shandong, China; ^3^ Institute of ornamental plants, Shandong Academy of Forestry, Jinan, Shandong, China

**Keywords:** *Paeonia lactiflora Pall.*, SLAF-seq, association study, population structure, DCAPS, marker-assisted selection

## Abstract

*Paeonia lactiflora Pall*. (*P. lactiflora*) is a famous ornamental plant with showy and colorful flowers that has been domesticated in China for 4,000 years. However, the genetic basis of phenotypic variation and genealogical relationships in *P. lactiflora* population is poorly understood due to limited genetic information, which brings about bottlenecks in the application of effective and efficient breeding strategies. Understanding the genetic basis of color-related traits is essential for improving flower color by marker-assisted selection (MAS). In this study, a high throughput sequencing of 99 diploid *P. lactiflora* accessions *via* specific-locus amplified fragment sequencing (SLAF-seq) technology was performed. In total, 4,383,645 SLAF tags were developed from 99 *P. lactiflora* accessions with an average sequencing depth of 20.81 for each SLAF tag. A total of 2,954,574 single nucleotide polymorphisms (SNPs) were identified from all SLAF tags. The population structure and phylogenetic analysis showed that *P. lactiflora* population used in this study could be divided into six divergent groups. Through association study using Mixed linear model (MLM), we further identified 40 SNPs that were significantly positively associated with petal color. Moreover, a derived cleaved amplified polymorphism (dCAPS) marker that was designed based on the SLAF tag 270512F co-segregated with flower colors in *P. lactiflora* population. Taken together, our results provide valuable insights into the application of MAS in *P. lactiflora* breeding programs.

## Introduction

The herbaceous peony (*P. lactiflora*) is an important ornamental plant, which is favored by consumers for various flower color. *P. lactiflora* have nine flower color categories, including red, pink, white, blue, purple, green, yellow, black, and double color (Wu et al., 2022). Anthocyanins and flavonoids are the main pigments in *P. lactiflora* flower (Zhao et al., 2012b). The types and contents of anthocyanins are responsible for pink and blue-purple in flowers, and the contents of flavonoids and flavonols are responsible for yellow flower (Dubos et al., 2008). The eight major anthocyanins in *P. lactiflora* flowers have been detected, including peonidin 3,5-di-*o*-glucoside (Pn3G5G), pelargonidin 3,5-di-*o*-glucoside (Pg3G5G), cyanidin 3,5-di-*o*-glucoside (Cy3G5G), peonidin 3-*o*-glucoside (Pn3G), cyanidin 3-*o*-glucoside (Cy3G), peonidin 3-*o*-gluco-side-5-*o*-arabino-side (Pn3G5Ara), cyanidin 3-*o*-glucoside-5-*o*-galactoside (Cy3G5Gal), and pelargonidin 3-*o*-glucoside-5-*o*-galacto-side (Pg3G5Gal) (Zhao et al., 2012b). The formation of *P. lactiflora* flower color is coordinated by many factors, including petal pigment, cell physiological environment, metal ion complexation and environmental factors (Guo et al., 2019). Therefore, the study on flower color can provide important theoretical support for improvement of *P. lactiflora* varieties and the cultivation of new varieties.

Flower color is one of the most valuable traits of ornamental plants, which is highly valued by researchers and producers. The scientific and accurate classification of flower color is of great significance to the study of flower color formation mechanism and variety classification. The CIELAB color space plays an important role in flower color measurement (Zhai et al., 2018). The colorimeter has advantages of good unity, high stability and reliability, which is not affected by the outside environment. The lightness (L^*^), redness (a^*^) and yellowness (b^*^) measured by a colorimeter can accurately describe the petal color (Gupta et al., 2005). Understanding the genetic basis of color-related traits (L^*^, a^*^, b^*^, C and ΔE) in *P. lactiflora* is a necessary condition for breeding new varieties using marker-assisted selection (MAS).

In several decades, with the continuous improvement of molecular biology methods, high-throughput and large-scale sequencing technologies have emerged, which has greatly reduced cost of sequencing (Ye et al., 2017a). Genome-wide association study (GWAS) has been widely used in crops, vegetables, ornamental plants, and so on, which has successfully located many genes related to agronomic traits (Chen et al., 2022). GWAS has the advantages of speed, accuracy and economy, which is favored by the breeders. SLAF-seq is specific-locus amplified fragment sequencing, which provides a efficient strategy for large-scale genotyping based on high-throughput sequencing (Sun et al., 2013a). SLAF-seq is a relatively new high-resolution strategy, which can quickly, accurately and efficiently develop large-scale InDel and SNPs markers (Liang et al., 2016). In consequence, SLAF-seq has laid a foundation for the wide application of GWAS, which will play a vital role in revealing the molecular mechanism of main agronomic traits formation in *P. lactiflora*. Therefore, it is of great significance to promote the transformation from traditional breeding to efficient and targeted molecular design breeding for *P. lactiflora* improvement.

Genetic diversity analysis is a key parameter for the identification and evaluation of germplasm resources, which plays an important role in mining genes related important agronomic traits and genetic breeding (Badaeva et al., 2022). The population structure, genetic relationship and genetic diversity are important indicators for population genetics research (Lu et al., 2019). With the development of modern molecular biotechnology, molecular markers are more widely used in population genetics research. For instance, genetic diversity analysis using SSR molecular markers resolved the genetic variation of different wild peonies at population and species levels (Xue et al., 2021). Population structure analysis based on SNPs showed that 122 *Osmanthus fragrans* accessions could be divided into 8 groups (Chen et al., 2021). There have been few studies on genetic diversity and population structure based on DNA molecular markers in *P. lactiflora*. Thus, study on genetic diversity, genetic relationship and population structure of *P. lactiflora* germplasm resources can reveal its genetic characteristics, which will provide reference for further study on the origin and evolution of *P. lactiflora*, and lay a foundation for germplasm identification, resource utilization and breeding.

With the rise of high-throughput sequencing technology, whole-genome sequencing has been widely used in many plants, which has successfully performed population structure analysis and located many genes related to agronomic traits (Cao et al., 2022). So far, this method has not been used to mine flower color-related SNPs in *P. lactiflora*. In order to provide theoretical and applied basis for MAS in early breeding of *P. lactiflora*, we aimed to use SLAF-seq-based association study to obtain SNPs related to flower color, thereby discovering favorable SNP variants and developing dCAPS markers that co-segregate with flower color, which can be used in MAS breeding in the future. To this end, by flow cytometry analysis, we selected 99 diploid varieties with more than 30 years cultivation from 159 *P. lactiflora* varieties for further analysis. Here, a high-through genome sequencing of 99 *P. lactiflora* accessions with different colors was conducted by SLAF-seq technology. Moreover, based on 2,954,574 SNPs from sequencing data, we analyzed population structure, genetic relationship and genetic diversity of *P.lactiflora* population, which suggested that this population could be divided into six subgroups. Furthermore, the favorable SNPs associated with flower color were identified in *P. lactiflora* population through association study. We developed peak SNP in 270512F fragment into dCAPS marker to assist early selection of color traits in *P. lactiflora*. In conclusion, this study laid a theoretical foundation for molecular breeding and genetic improvement of important traits in *P. lactiflora*.

## Materials and methods

### Plant materials and growth conditions

A total of 159 P*. lactiflora* cultivars were collected from Shandong province, China, including 34 P*. lactiflora* accessions from Jinan city (36°35′~36°40′ N, 116°54′~117°02′ W), 81 P*. lactiflora* accessions from Heze city (34°39′~35°52′ N, 114°45′~116°25′ W), and 44 P*. lactiflora* accessions from Tai’an city (35°38′~36°28′ N, 116°20′~117°59′ W). Based on flower color, 159 P*. lactiflora* accessions were divided into four groups, including white group, pink group, multicolor group and purple group (**Supplementary Figure 1**). Of them, the representative 99 *P. lactiflora* varieties with more than 30 years cultivation identified as diploid were used for population structure analysis and association study based on flower color variation of population in this study. Also, the F_1_ population consisting of 14 individuals resulting from the cross between cultivated *P. lactiflora* “Zifengyu” with purple flower and “Fenghuangniepan” with white flower was grown in open-field cultivation at Shandong Agricultural University.

### Determination of nuclear DNA ploidy by flow cytometry

The 159 *P. lactiflora* cultivars were collected for nuclear DNA ploidy analysis in order to obtain diploid cultivars. The mature leaves were chopped with a blade in 400 μL ‘Aru’ Buffer and then filtered into 2 mL PE tube with a 40 μm filter. The samples were stained with propidium iodide and RNA enzyme for 5 min and then measured at 561 nm using BD flow cytometry (BD Biosciences, San Jose, California, USA). The sample was loaded at a medium speed. We selected diploid cultivars ‘Linghuazhanlu’ that were closely related to *P. lactiflora* as reference, and then identified 143 diploid *P. lactiflora* cultivars (**Supplementary Figures 2**–**4**).

### Outer-petal color measurement

CIELAB was used to measure color parameters of *P. lactiflora* flower. The color-related values (L^∗^, a^∗^ and b^∗^) of fresh petals at bloom stage were determined by a colorimeter (CR-400, Konica Minolta, Osaka, Japan). Chroma (C), hue angle (h), and total color-difference (ΔE) were calculated according the described equations: C=(a^∗2^+b^∗2^)^1/2^, h=a^∗^/b^∗^, and ΔE =(L*^2^+a*^2^+b*^2^)^1/2^, respectively (Lee, 2005; Yang et al., 2020a). In the uniform color space, L^*^ represented the lightness, a^*^ represented the ratio of red and green, b^*^ represented the ratio of yellow and blue, and H represented hue angle (Zhao et al., 2012a). ΔE can accurately reflect the accuracy of the color (Gregor et al., 2016). The 1, 2, 3, and 4 represented yellow, white, pink, and purple, respectively. The color parameters of flowers at bloom stage were counted for 159 *P. lactiflora* accessions in the spring of 2020, 2021 and 2022. The trials of three consecutive years were considered as three independent replicates, and each biological replicate contained three individual flowers from the same genotype. Three-year data of outer-petal color of flowers in each accession were averaged for subsequent association study.

### SLAF sequencing and SNP calling

We used the cetyltrimethylammonium bromide strategy to extract genomic DNA from young leaves of each *P. lactiflora* cultivar. Then, HinCII and EcoRV enzymes were identified to digest genomic DNA of every accession. Ultimately, SLAF-seq was performed by using an Illumina Hiseq 2500 (Illumina Inc; San Diego, CA, USA) at Biomarker Technologies Corporation in Qingdao, China. The guanine−cytosine (GC) content and Q30 were used to estimate sequencing quality after removing adapter reads (*Q* =−10×log _10_^e^, indicating 99.9% confidence level and a 0.1% error probability). A total of SNPs was identified *via* GATK and SAM tools programs based on the polymorphic SLAF tag information (Sun et al., 2013).

### Population genetics analyses

Based on reliable 2,954,574 SNPs developed from 99 P*. lactiflora* accessions through SLAF-Seq, we performed population structure, phylogenetic tree and principal component analysis (PCA). The population structure was estimated with ADMIXTURE program. The phylogenetic tree was finished with the neighbor-joining strategy in MEGA5. GAPIT and ADMIXTURE program were used to perform the population structure and PCA, respectively (Alexander et al., 2009).

### Association study

We used 722,339 high quality SNPs to perform association study for flower color in 99 *P. lactiflora* accessions using the *Paeonia suffruticosa* genome as a reference genome. The association study was conducted using the MLM with TASSEL4.0. The *P* value of each SNP was calculated and significance was defined at a uniform threshold of < 1.38×10^−6^ (*P* =1/n, where n was the total number of SNPs in this association analysis). We performed association study using previously reported method (Bradbury et al., 2007).

### dCAPS marker for flower color

The SNP in 270512F fragment from association study was suitable for conversion to dCAPS marker and we selected the restriction enzyme *SspⅠ* (NEB) using dCAPS Finder 2.0 (http://helix.wustl.edu/dcaps/dcaps.html). A pair of primers flanking the SNP locus in 270512F fragment was designed by PRIMER PREMIER 5.0. A 536-bp fragment of 270512F sequence was amplified from each *P. lactiflora* cultivar using the following primers: a forward primer, 5’- AACTAAATCCAATCCAACATGAAAATGGTTTTTACACTAGCCCAGGCACACCCAACAATTTCAACAAACAAAAAAAATGTCAAGGAATAT-3’, and a reverse primer, 5’-CATTGAGAGGCCACCATGATTAA-3’. The PCR program was performed as follows: (1) 3 min at 94°C; (2) 34 cycles of 30 s at 94°C, 30 sat 56°C and 50 s at 72°C; (3) 10 min at 72°C. The PCR products were subsequently digested with *SspⅠ* for 2 h at 37°C in 10 μL reaction volumes containing 3 μL PCR products, 1 μL 10 х buffer, 0.3 μL *SspⅠ* (5 units/mL), and 5.7 μL double-distilled water. DNA products after enzymatic digestion were then separated by electrophoresis in 1% agarose gels stained with GelRed and displayed *via* UV light.

## Results

### Variation analysis of flower color phenotypes among 159 *P. lactiflora* accessions

**Figure 1:**
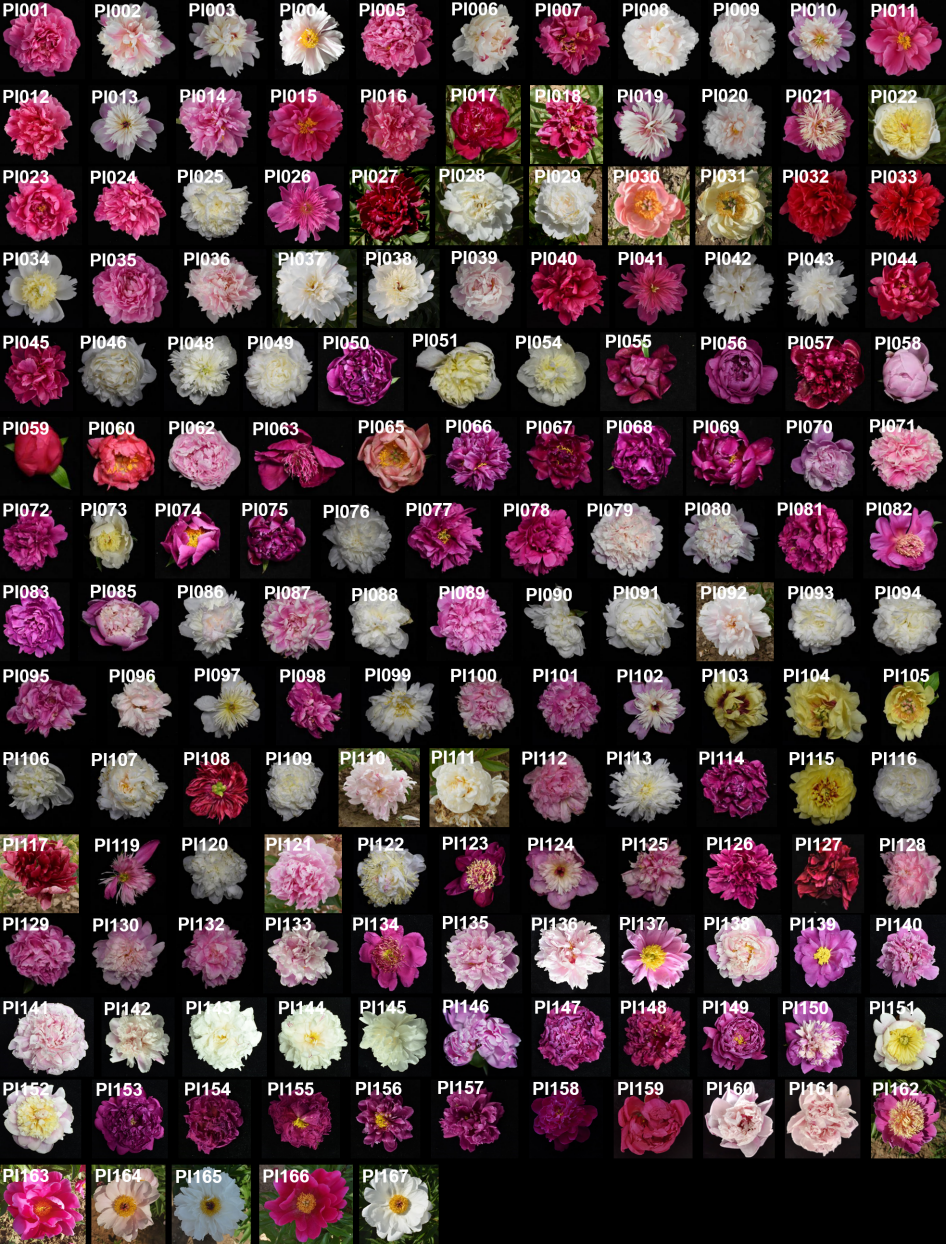
Flowers of 159 *P. lactiflora* accessions in bloom stage.

**Figure 2:**
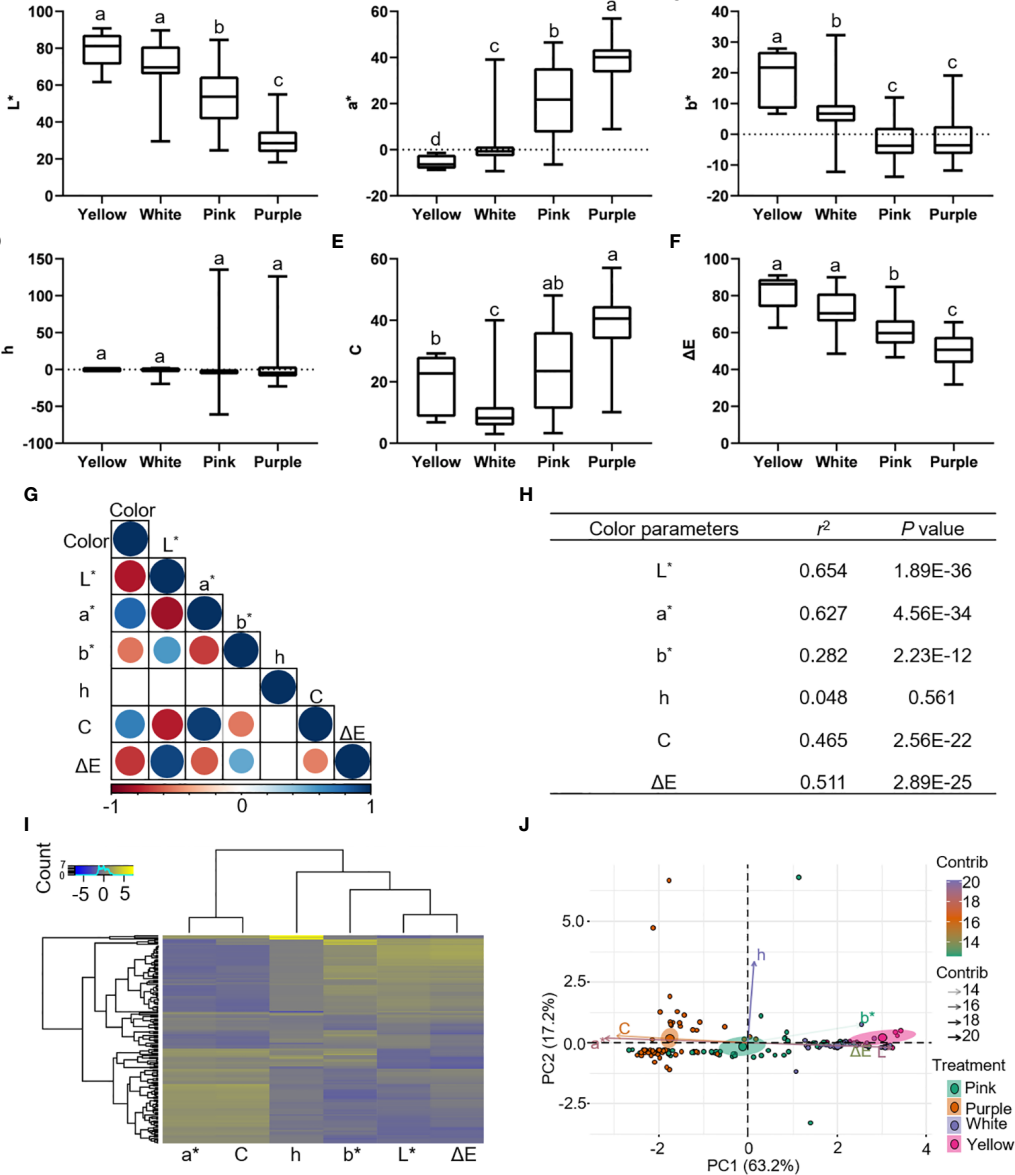
Color parameters of 159 *P. lactiflora* accessions. **(A–F)** Distribution of color indices in yellow, white, pink and purple groups. L^*^, a^*^, b^*^, h, C and ΔE represent the brightness, redness, yellowness, hue angle, chroma and hue, respectively. Results are expressed as means ± SD (n = 6). Different letters indicate significant differences at *p* < 0.05 according to Duncan’s new multiple-range test; ANOVA, analysis of variance. **(G, H)** Correlation analysis between color and color parameters. Clustering analysis **(I)** and principal component analysis **(J)** were used to analyze color parameters of 159 *P. lactiflora* samples. R programming is used for data analysis.

To examine whether significant phenotypic variances exist in the flower colors among 159 P*. lactiflora* accessions, the six color-related traits including L^∗^, a^∗^, b^∗^, h, C and ΔE were determined using the CIELAB system (**Supplementary Tables 1**–**8**). The results showed that the L^∗^ (brightness) value ranged from 18.14 to 90.79, a^∗^ (redness) from -9.28 to 56.90, b^∗^ (yellowness) from -13.8 to 32.29, C value ranged 3.03 from to 57.06, and ΔE value ranged from 31.86 to 91.05. Phenotypic variation analysis of flower color showed that the indices of flower colors from different accessions were quite diverse (**Supplementary Table 9**). The 159 *P. lactiflora* accessions according to their flower color were divided into four categories including white group, pink group, purple group and multicolor group (**Figure 1**). The color-difference value analysis of external petal color showed that L^*^ and ΔE decreased from yellow petals to purple petals (**Figures 2A, F**). However, a^∗^ and C increased from yellow petals to purple petals (**Figures 2B, E**). The b^∗^ values of white petals were higher than pink and purple petals (**Figure 2C**). There was no significant difference in h value among the three-color petals (**Figure 2D**).

In order to better understand the relationship between petal color and indices based on CIELAB system, we performed correlation analysis to reveal that L^∗^, a^∗^, C and ΔE had high significant with color, and their *Pearson*’s correlation coefficients were 0.654, 0.627, 0.465, and 0.511, respectively (**Figures 2G, H**). Interestingly, the clustering analysis and principal component analysis showed that color indices based on CIELAB system can distinguish different color petals, which can well reflect the color of the petals (**Figures 2I, J**). Overall, these analyses indicated that variant-rich *P. lactiflora* population is eligible for GWAS analysis.

### Genome sequencing and SNP genotyping of 99 *P. lactiflora* accessions

**Figure 3:**
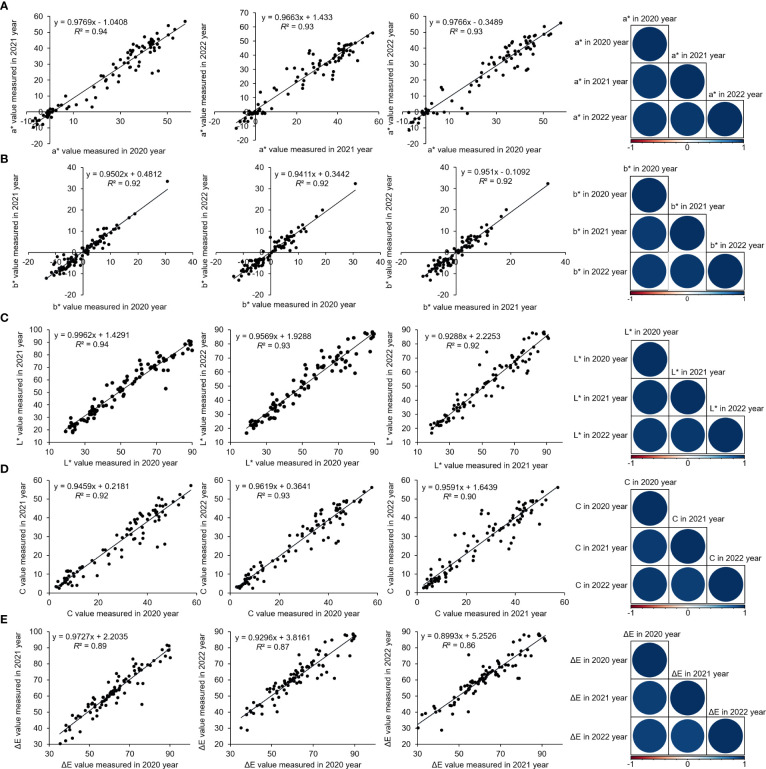
Correlation between flower color parameters of 99 *P. lactiflora* accessions over the three years (2020, 2021, and 2022). Linear regression analysis of a* **(A)**, b* **(B)**, L* **(C)**, C **(D)**, and ΔE **(E)**. R programming is used for data analysis.

Polyploid *P. lactiflora* varieties had more complex genome than diploid varieties, which might hinder the association study. We first performed ploidy analysis of all 159 varieties using flow cytometer and isolated 143 diploid *P. lactiflora* varieties. We chose 99 out of 143 diploid varieties for population structure analysis and association analysis since they were traditional varieties cultivated for more than three decades. However, the population size is relatively small and may be only suitable for the identification of major genetic loci (Atwell et al., 2010). We then applied the coefficient of variation combined with the Shannon-Wiener index to evaluate the variation and genetic diversity of color-related traits in the *P. lactiflora* population, which revealed a broad diversity in color parameters in the population of the 99 *P. lactiflora* accessions (**Supplementary Figure 5**). Linear regression of three-year phenotypic data revealed a strong linear correlation between the *P. lactiflora* flower color-related traits, implying that the phenotypic variation of *P. lactiflora* flower color was mainly derived from genetic factors (**Figure 3**).

We sequenced the 99 diverse *P. lactiflora* accessions by the SLAF-seq method based on HinCII and EcoRV digestion and altogether obtained 737.00 Mb pair-end reads with 126-bp read length (**Table 1** and **Supplementary Table 10**). Q30 represents the quality score of 30, which indicates that the sequence accuracy is 99.9% (Xie et al., 2018). The average value of Q30 of 99 P*. lactiflora* accessions was 92.48%, and average GC (guanine-cytosine) content was 37.09% (**Supplementary Table 11**). Altogether, we developed 4,383,645 SLAF tags from all accessions with an average sequencing depth of ~20.81× for each SLAF tag (**Supplementary Table 12**). A total of 2,954,574 SNPs (MAF ≥ 0.05) were obtained from all SLAF tags. And, most of the accessions had a lower heterozygosity ratio (**Supplementary Table 13**). These results showed that polymorphic SLAF tags and SNPs were evenly distributed in 99 *P. lactiflora* accessions, indicating that the sequencing results are reliable and can be used for further population structure and association study.

**Table 1:** Summary of statistic data generated by SLAF-seq technology.

Samples	Total Reads	Total SLAF tags	Average depth of SLAF tags	Polymorphic SLAF tags	Total SNPs
99	737.00 Mb	4,383,645	20.81x	136,377	2,954,574

### Phylogenetic and population structure analysis of *P. lactiflora* population

**Figure 4:**
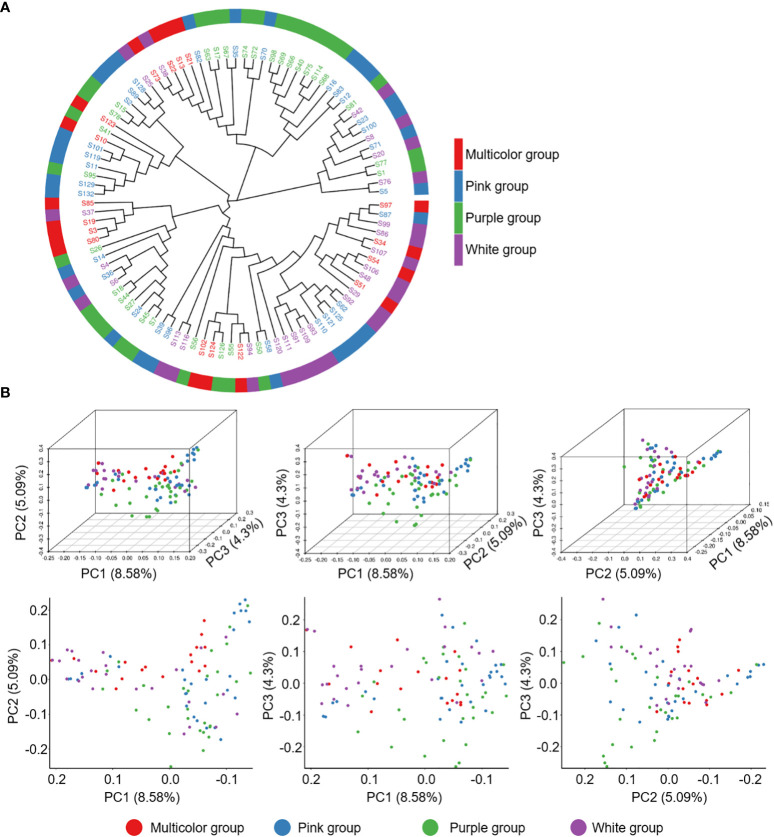
Population genetics of 99 *P. lactiflora* accessions. **(A)** The phylogenetic tree was constructed using Kimura 2-parameter model. Accessions with the same color belong to the same group. **(B)** Principal components analysis of 99 P*. lactiflora* accessions. Each dot represents an accession.

To understand the genotype divergence in a collection of 99 *P. lactiflora* accessions, population structure, phylogenetic tree and principal component analysis (PCA) were performed using the total SNPs. The population structure analysis from K = 1 to 10 was calculated with Admixture software (**Supplementary Figure 6A**). The delta K value reached the lowest point at K = 6, revealing that six population clusters represented the optimal model (**Supplementary Figure 6B** and **Supplementary Table 14**). We analyzed the petal colors of accessions in each cluster and did not observe the correlation between petal color and cluster. Meanwhile, we could not subjectively divide the petal color into six groups because of too many color variations, which prompted us to divide 99 accessions into four groups including white, purple, pink and multicolor and ensure the clear separation of extreme color groups. The MEGAX software based on neighbor-joining phylogeny was further used to calculate the phylogenetic relationships of *P. lactiflora* population. *P. lactiflora* population could be divided into 6 divergent groups, which was were largely consistent with the results of group structure (**Figure 4A**). To further support the result, a PCA was performed using EIGENSOFT software (**Supplementary Table 15**). The first three dimensions of principal components interpreted 8.58% (PC1), 5.09% (PC2), and 4.30% (PC3) of the genetic variation, respectively (**Figure 4B**). Genetic diversity analysis revealed that genetic variation within groups was abundant and the genetic diversity between groups was similar (**Table 2**). Hence, this *P. lactiflora* population for association study was relative ideal.

**Table 2:** Genetic diversity analysis of four *P. lactiflora* groups based on SNPs.

Group	Average MAF	Expected allele number	Expected heterozygous number	Nei diversity index	Number of poly marker	Observed allele number	Observed heterozygous number	Polymorphysm information content	Shnnon Wiener index
MT	0.2274	1.475	0.2869	0.2973	167360	1.915210675	0.2243	0.2328	0.4389
PK	0.2194	1.4918	0. 2995	0.3055	179405	1.981078938	0.2336	0.2439	0.4605
PP	0.221	1.4941	0.3004	0.3063	178958	1.978634512	0.2455	0.2444	0.4612
WT	0.2238	1.491	0.2978	0.3049	175544	1.959965002	0.2344	0.2421	0.4566

MT, multicolor group; PK, pink group; PP, purple group; WT, white group.

### Association analysis of color-related traits by MLM model

**Figure 5:**
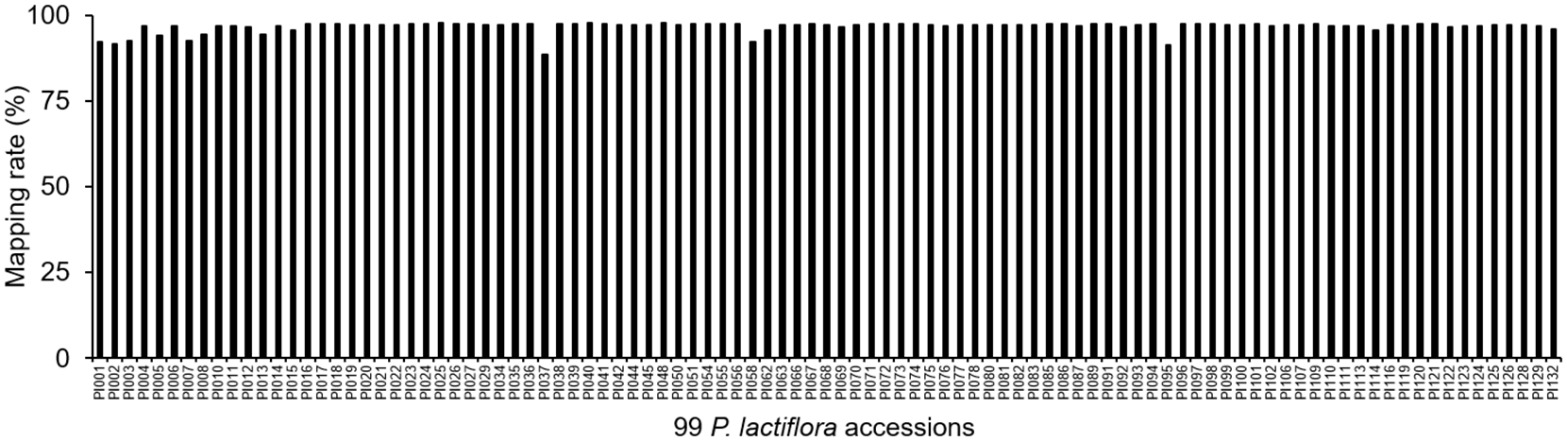
Mapping rates of reads from 99 *P. lactiflora* accessions mapped to *Paeonia suffruticosa* genome.

We mapped the reads obtained from 99 *P. lactiflora* varieties by SLAF-Seq to *Paeonia suffruticosa* genome to obtain the mapping rates (**Supplementary Table 16**). The average mapping rate of the *P. lactiflora* population was 96.70%, which indicated that the genomes of *P. lactiflora* and *Paeonia suffruticosa* had high identities (**Figure 5**). These results suggest that the *Paeonia suffruticosa* genome can be used as a reference for subsequent association analysis and marker development of the *P. lactiflora*.

**Figure 6:**
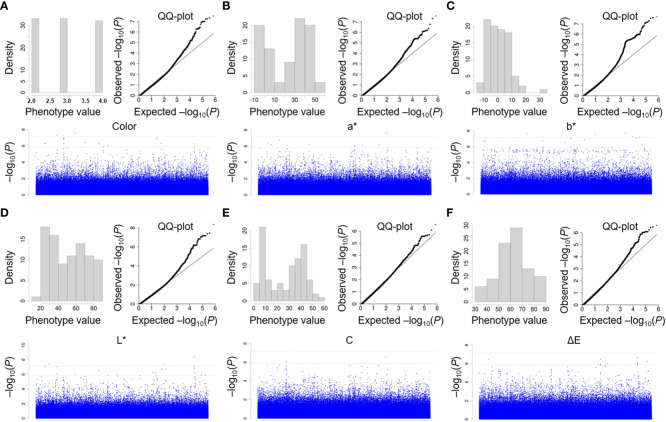
Association study of color indices in *P. lactiflora* population using MLM model. Manhattan plots of association study by MLM model for color **(A)**, a^*^ **(B)**, b^*^ **(C)**, L^*^ **(D)**, C **(E)** and ΔE **(F)**, respectively. The test threshold is shown as a dash black line (at *P* < 0.01 and *P* ≤ 0.05). Each SNP is marked by a dot.

There was abundant variation in the flower color of *P. lactiflora* at the bloom stage. To uncover and evaluate the genetic variants associated with flower color, the MLM for an association study was performed to analyze and identify the genetic loci of those traits. We chose *Paeonia suffruticosa* genome as reference genome for association study (**Figure 6**). After filtering, reads generated from 99 P*. lactiflora* accessions were aligned to the *Paeonia suffruticosa* reference genome. Then, we obtained high quality 722,339 SNPs. Also, we performed QQ plots to evaluate the extent of accordance between observed and expected *P* values. MLM analysis detected 8, 5, 9, 11, 1 and 6 SNPs significantly associated with color, a^*^, b^*^, L^*^, C and ΔE at *P* < 1.38 × 10^−6^, respectively. Totally, 40 SNPs significantly associated with six traits were detected by association study (**Table 3**).

**Table 3:** SNP loci significantly associated with color traits in 99 *P. lactiflora* accessions.

Trait	SNP	Chr. (fragment)	Position	*P* value	Allele 1	Allele 2	Trait	SNP	Chr. (fragment)	Position	*P* value	Allele 1	Allele 2
Color	016669F_93937	016669F	93937	2.83E-08	C	T	b*	107146F_12389	107146F	12389	1.27E-06	T	C
Color	008589F_22390	008589F	22390	4.67E-08	C	T	b*	099778F_13298	099778F	13298	1.28E-06	A	C
Color	011971F_70287	011971F	70287	5.69E-08	A	T	L*	121822F_29251	121822F	29251	4.27E-08	G	T
Color	011971F_70338	011971F	70338	1.02E-07	C	A	L*	2666578F_6595	266578F	6595	6.68E-08	T	C
Color	102917F_9224	102917F	9224	1.13E-07	C	T	L*	008589F_22390	008589F	22390	9.03E-08	C	T
Color	022420F_28194	022420F	28194	3.62E-07	G	A	L*	116100F_11768	116100F	11768	1.45E-07	G	T
Color	032062F_38959	032062F	38959	5.33E-07	C	T	L*	127289F_253	127289F	253	2.1E-07	G	C
Color	266578_6817	266578F	6817	1.33E-06	C	A	L*	032062F_38959	032062F	38959	2.93E-07	C	T
a*	121822F_29251	121822F	29251	2.62E-08	G	T	L*	094781F_10951	094781F	10951	5.47E-07	A	C
a*	008589F_22390	008589F	22390	1.75E-07	C	T	L*	100900F_11506	100900F	11506	6.54E-07	C	T
a*	102917F_9224	102917F	9224	7.19E-07	C	T	L*	270512F_4769	270512F	4769	6.78E-07	G	T
a*	073661F_45161	073661F	45161	8.17E-07	G	A	L*	016669F_93937	016669F	93937	1.04E-06	C	T
a*	127289F_253	127289F	253	1.36E-06	G	C	L*	381852F_3833	381852F	3833	1.34E-06	G	C
b*	020676F_85589	020676F	85589	2.47E-08	A	G	C	121822F_29251	121822F	29251	2.65E-07	G	T
b*	229915F_515	229915F	515	4.91E-08	T	C	ΔE	103536F_9221	103536F	9221	3.35E-07	G	T
b*	075585F_40827	075585F	40827	1.38E-07	C	A	ΔE	363352F_1667	363352F	1667	5.94E-07	G	T
b*	001349F_1122739	001349F	1122739	1.71E-07	G	T	ΔE	266578F_6793	266578F	6793	8.43E-07	C	T
b*	031372F_57833	031372F	57833	1.82E-07	C	T	ΔE	121822F_29251	121822F	29251	9.71E-07	G	T
b*	050466F_9656	050466F	9656	2.71E-07	C	T	ΔE	266578F_6548	266578F	6548	1.24E-06	G	A
b*	001364F_34932	001364F	34932	3.12E-07	G	A	ΔE	100900F_11506	100900F	11506	1.32E-06	C	T

### Development and verification of 270512F−based dCAPS marker

**Figure 7:**
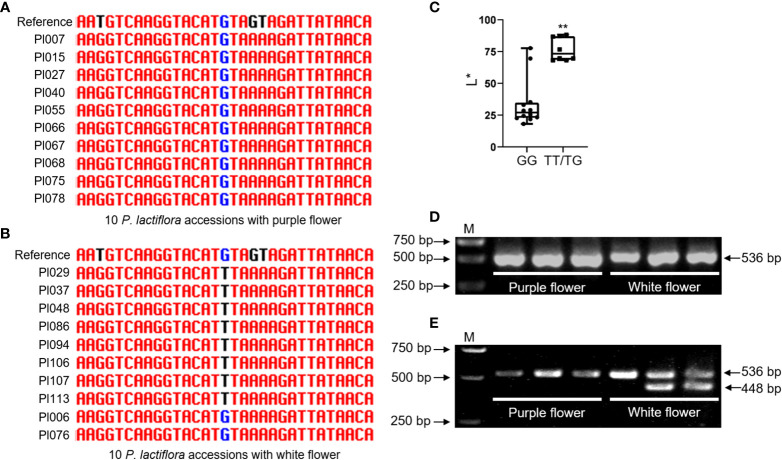
Genotyping of different *P. lactiflora* cultivars using a 270512F_4769-based dCAPS marker. **(A)** Amplification of 270512F-based fragment in 10 *P. lactiflora* cultivars with purple flower, randomly. **(B)** Amplification of 270512F-based fragment in 10 P*. lactiflora* cultivars with white flower, randomly. **(C)** Box plot for the L^*^ of individuals with different SNP allele. Each dot represents an individual. **(D)** PCR products of three randomly selected cultivars with purple flower and three randomly selected cultivars with white flower using the dCAPS primer. **(E)** Polymorphism analysis of dCAPS marker in individuals with different colors. **Significant difference at *P* < 0.01, as calculated by Student’s *t* test. M represents DNA marker.

The CAPS or dCAPS markers based on significant trait-associated SNP loci were designed to accelerate the molecular breeding of ornamental plants (Lu et al., 2017). The L^*^ value can represent the brightness of the flower, which is most closely related to flower color (**Figure 2H**). The 270512F that explained the highest SNP of L^*^ for external petal color *via* association study was selected for marker development (**Figure 6D** and **Supplementary Table 17**). We randomly selected 10 varieties with white flower and 10 varieties with purple flower from 99 *P. lactiflora* accessions to amplify 270512F partial fragment by PCR. Significantly, a SNP in this fragment was linked to flower color by Sanger sequencing (**Figures 7A, B**). The L^*^ value of varieties with TT/TG allele was significantly greater than those with GG allele (**Figure 7C**). To develop specific SNP-based marker, we introduced two mismatches into the forward primer to design the specific PCR-based dCAPS marker. As a result, a pair of dCAPS marker primer was developed using the restriction enzyme *SspⅠ*. The fragment size of the PCR product was 536 bp for 270512F−based dCAPS. Two bands of 448 bp and 88 bp were produced in the PCR products from varieties with white flower after *SspⅠ* digestion, whereas varieties with purple flower could not be digested and remained only one band of 536 bp (**Figures 7D, E**). In general, the results showed that the co-segregation ratio of the 270512F−based dCAPS marker and flower color was up to 80% in *P. lactiflora* population.

## Discussion

### Genome sequencing of *P. lactiflora via* SLAF-Seq provided high-quality SNPs for further analysis

*P. lactiflora*, commonly known as the herbaceous peony, is a well-known ornamental plant that possesses the extremely abundant flower colors and shapes, which is favored by worldwide people (Wang et al., 2020). However, *P. lactiflora* has unsequenced genome and an incompletely clear genetic diversity, which severely limits its in-depth study. SLAF-Seq is a high-throughput sequencing technology that can easily and quickly obtain SNP markers and genotypes covering the whole genome, which has characteristics of simple and rapid library construction, low cost, high accuracy, and so on (Liu et al., 2018). SLAF-Seq can be widely used in genetic diversity analysis and the GWAS analysis with reference genome and non-reference genome (Huang et al., 2021). In order to better carry out the molecular research of *P. lactiflora*, we performed SLAF-Seq and obtained high-quality 2,954,574 SNPs for further analysis.

### Genetic diversity explained that *P. lactiflora* population were clustered into six groups

To study the genetic diversity that can provide valuable information for breeding and germplasm innovation, we performed SLAF-seq on 99 *P. lactiflora* accessions. Since germplasm resources are the basis of breeding work, it is very important to clarify the genetic diversity, genetic background and genetic structure of varieties for breeding (Zhang et al., 2021). In recent years, the population structure of some species has been explored using genome sequencing on the basis of SLAF-seq (Chen et al., 2018). Previous reports indicated that flower color of ornamental plants as an important phenotypic trait had always been an important basis for its population structure analysis (Chen et al., 2021a). Due to the *P. lactiflora* genome has not been sequenced, the traditional morphological classification of *P. lactiflora* population is based on phenotype of flower color. Meaningfully, our results of the population structure and phylogenetic tree analysis based on genome sequencing of 99 *P. lactiflora* accessions showed that *P. lactiflora* population were clustered into six groups (**Figure 4** and **Supplementary Figure 6**). Therefore, we develop a large genome variation data set for genetically diverse *P. lactiflora* accessions, which helps guide the genetic improvement and resource conservation of *P. lactiflora*.

### The color of *P. lactiflora* flowers was a complex quantitative trait

The petal colors of *P. lactiflora* range from purple, red, pink, white and rarer colors, which implies that color is a complex trait. Furthermore, we investigated flower color phenotype of the F_1_ population composed of 14 individuals developed from the cross between cultivated *P. lactiflora* ‘Zifengyu’ with purple flower and ‘Fenghuangniepan’ with white flower and found 5 purple individuals, 8 pink individuals and one white individual (**Supplementary Figure 7**). These data suggested that flower color of *P. lactiflora* is a quantitative trait, in which purple and white traits were controlled by dominant and recessive genes, respectively. In addition, the coefficient of variation (CV) and Shannon-Wiener index can be used to evaluate the phenotypic variation of population (Döring and Reckling, 2018; Wei et al., 2019). Variance analysis of *P. lactiflora* petal colors exhibited a broad diversity in *P. lactiflora* population. The many important traits of *P. lactiflora* are controlled by multiple genes and are easily affected by environment factors, which is difficult to select these traits by conventional breeding approaches (Yang et al., 2020b). Therefore, it is necessary to develop molecular markers related to important traits for the genetic improvement of *P. lactiflora*.

### Identification of SNP loci associated with traits related to flower color in *P. lactiflora*

To date, GWAS is used to explore the relationship between natural allelic variations and phenotypic variation and has been widely considered effective approach to detecting and identifying SNPs responsible for complex quantitative traits (Liu et al., 2020). SLAF-seq-based GWAS were employed to obtain 1,392,755 high-quality SLAF tags and discover 26 SNP loci related to the timing of spring bud flush of tea (*Camellia sinensis*) (Wang et al., 2019). *P. lactiflora*, belonging to *Paeonia*, has close genetic relationship with *Paeonia suffruticosa* (Wu et al., 2021). A *de novo* assembly and annotation of *Paeonia suffruticosa* genome have drafted using the PacBio’s Single Molecule, Real-Time Technology, which lays the foundation for functional genomics research of *Paeonia* plants (Lv et al., 2020). Although *P. lactiflora* genome has not been sequenced, *Paeonia suffruticosa* genome could be used as a reference genome for association study of *P. lactiflora*. Indeed, these GWAS methods with reference genomes are more powerful and accurate compared to GWAS methods without reference genomes (Guo et al., 2021). Therefore, to better demonstrate the significant SNPs associated with favorable variants, we adopted *Paeonia suffruticosa* genome as a reference for association study of *P. lactiflora* in this study. We identified 40 major polymorphic loci associated with flower color of *P. lactiflora* (**Table 3**). We are going to collect more *P. lactiflora* germplasms to optimize the population structure. In addition, the validation of these candidate SNPs in *P. lactiflora* hybrid progenies will be needed to provide further support for these conclusions.

### dCAPS marker effectively distinguished varieties with purple and white flowers

The MAS is a breeding technology that uses genetic markers closely linked to target phenotypic trait to track and select the target traits, which has been widely developed and applied in many crops (Ye et al., 2017). Compared with traditional breeding approaches, this MAS can accelerate the breeding process in view of early selection in a molecular breeding process. However, MAS technology has not been reported in *P. lactiflora*. In the research, we performed the SLAF-seq-based association study for flower color in 99 *P. lactiflora* accessions to discover 40 SNPs responsible for flower color (**Table 3**). Among these loci, a SNP locus in 270512F fragment significantly associated with flower color of *P. lactiflora* was designed into a dCAPS marker, which was codominant and locus-specific (**Figure 7**). In future studies, we will construct a F_2_ population to investigate the segregation of the developed SNP locus as well as for trait selection. Also, this developed dCAPS marker should be validated in larger populations with different genotype. Overall, the dCAPS marker co-segregated with flower color can assist in the selection of flower color traits of *P. lactiflora* at the seedling stage and offered a powerful breeding tool in MAS.

In this study, SLAF-Seq analysis based on the high throughput sequencing technology provided an efficient approach for analyzing of population structure and identifying SNPs responding to flower color in *P. lactiflora*. The population structure analysis showed that these 99 *P. lactiflora* accessions are clustered into six groups. Furthermore, we performed association study using MLM analyses based on color-related traits of 99 *P. lactiflora* cultivars, and ultimately detected 40 SNPs significantly associated with flower color. Importantly, one major polymorphic locus in 270512F demonstrating relatively strong association with flower color was excavated in 99 *P. lactiflora* accessions, and a dCAPS marker based on 270512F was designed that co-segregated with flower color, which could be helpful for future MAS breeding. Taken together, the elite SNPs identified according to flower color in the *P. lactiflora* can be directly applied to the targeted breeding of flower color.

## Conclusions

In this study, a total of 737.00 Mb reads, the 136,377 polymorphic SLAF tags and 2,954,574 SNPs were obtained based on SLAF-seq. We analyzed the population structure and genetic diversity of 99 P*. lactiflora* accessions and identified six independent genetic groups. We retrieved a large number of SNPs in these accessions, and 40 SNPs were significantly associated with color-related traits. We developed dCAPS markers based on the SLAF tag 270512F, which was 80% co-segregated with flower color in *P. lactiflora* population (**Figure 7**). In summary, our results provide new insights into the genetic basis of flower colors and will accelerate biotechnology assisted *P. lactiflora* breeding.

## Data availability statement

The data presented in the study are deposited in the NCBI repository, accession number PRJNA881230.

## Author contributions

FM, ZB and JC conceived and designed the experiments. GL and YL performed most of experiments. GL and YL analyzed the data. GL, FM and ZB wrote the manuscript. XS and XG supplied the experimental materials. NJ and YF provided the technical support. GL and YL contributed equally to this work. All authors contributed to the article and approved the submitted version.

## Funding

We gratefully acknowledge the support of Shandong province improved seed project (2020LZGC011-1 to FM).

## Acknowledgments

We thank College of Horticulture Science and Engineering at Shandong Agricultural University for supporting the experimental core facilities where we performed mass spectrometry and phenotypic analysis. We thank State Key Laboratory of Crop Biology at Shandong Agricultural University for providing lab space and plant growth chambers. We thank Mr. Qian Zhang, Mr. Zhiwei Wang, and Mr. Haijun Zhao for their technical support.

## Conflict of interest

The authors declare that the research was conducted in the absence of any commercial or financial relationships that could be construed as a potential conflict of interest.

## Publisher’s note

All claims expressed in this article are solely those of the authors and do not necessarily represent those of their affiliated organizations, or those of the publisher, the editors and the reviewers. Any product that may be evaluated in this article, or claim that may be made by its manufacturer, is not guaranteed or endorsed by the publisher.
