# Supplementary material for: Association study of SNP locus for color related traits in herbaceous peony (Paeonia lactiflora Pall.) using SLAF-seq
Source: Front Plant Sci. 2022 Dec 5;13:1032449. doi: 10.3389/fpls.2022.1032449 (PMC9760751; doi:10.3389/fpls.2022.1032449)
Supplement: Supplementary file 1 [file DataSheet_1.pdf]

Supplementary Figures 1-7

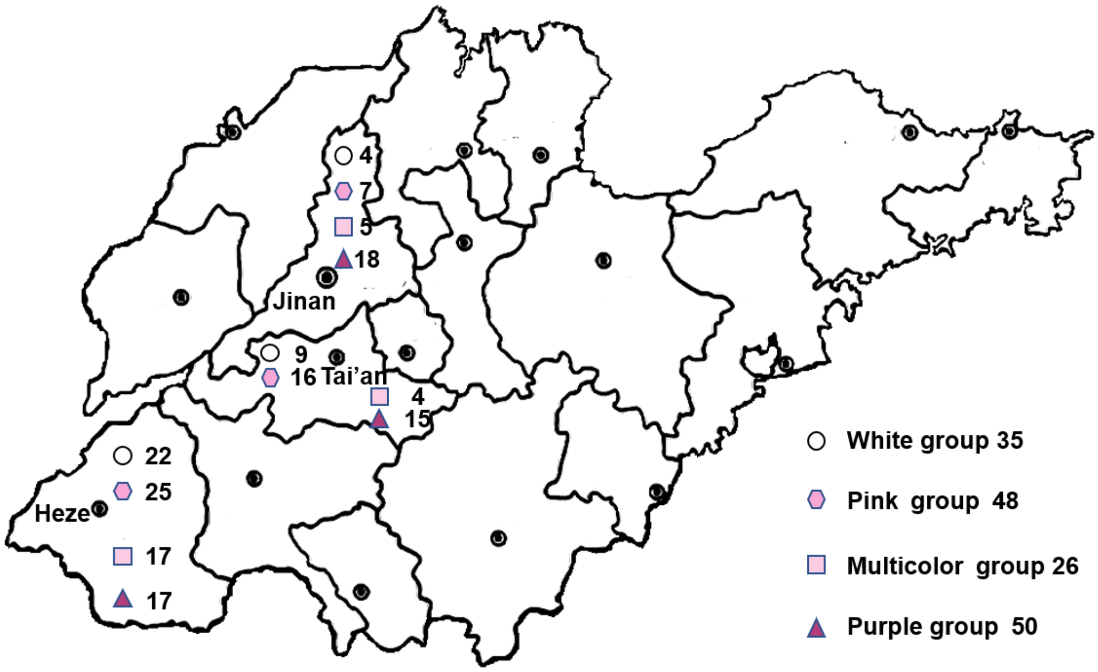

Supplementary Figure 1. The geographical distribution of 159 *P. lactiflora* accessions in Shandong province of China.

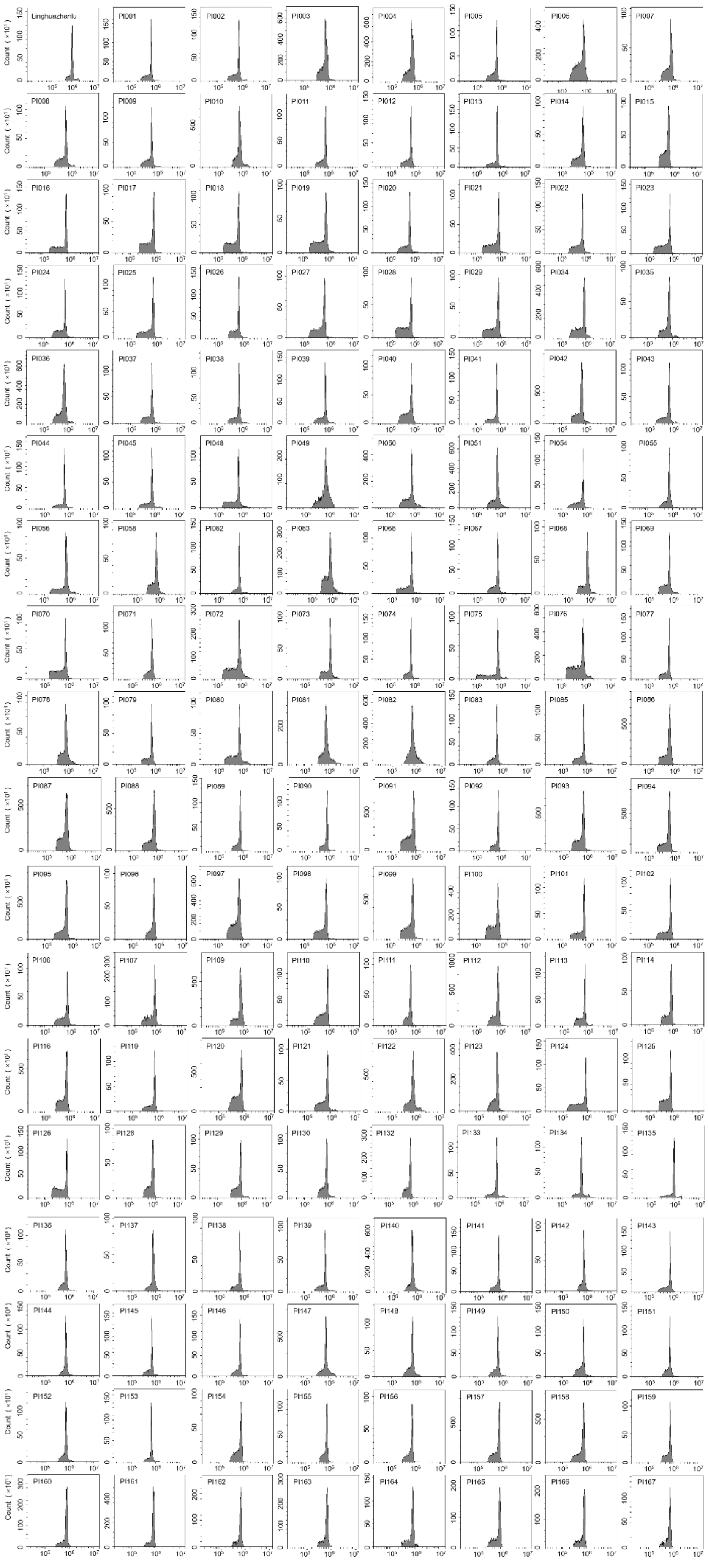
Count (×10³)
Linghuazhanlu
PI001
PI002
PI003
PI004
PI005
PI006
PI007
PI008
PI009
PI010
PI011
PI012
PI013
PI014
PI015
PI016
PI017
PI018
PI019
PI020
PI021
PI022
PI023
PI024
PI025
PI026
PI027
PI028
PI029
PI034
PI035
PI036
PI037
PI038
PI039
PI040
PI041
PI042
PI043
PI044
PI045
PI048
PI049
PI050
PI051
PI054
PI055
PI056
PI058
PI062
PI063
PI066
PI067
PI068
PI069
PI070
PI071
PI072
PI073
PI074
PI075
PI076
PI077
PI078
PI079
PI080
PI081
PI082
PI083
PI085
PI086
PI087
PI088
PI089
PI090
PI091
PI092
PI093
PI094
PI095
PI096
PI097
PI098
PI099
PI100
PI101
PI102
PI106
PI107
PI109
PI110
PI111
PI112
PI113
PI114
PI116
PI119
PI120
PI121
PI122
PI123
PI124
PI125
PI126
PI128
PI129
PI130
PI132
PI133
PI134
PI135
PI136
PI137
PI138
PI139
PI140
PI141
PI142
PI143
PI144
PI145
PI146
PI147
PI148
PI149
PI150
PI151
PI152
PI153
PI154
PI155
PI156
PI157
PI158
PI159
PI160
PI161
PI162
PI163
PI164
PI165
PI166
PI167

Supplementary Figure 2. Flow cytometric analysis of 143 diploid *P. lactiflora* varieties using diploid cultivar 'Linghuazhanlu' as a reference.

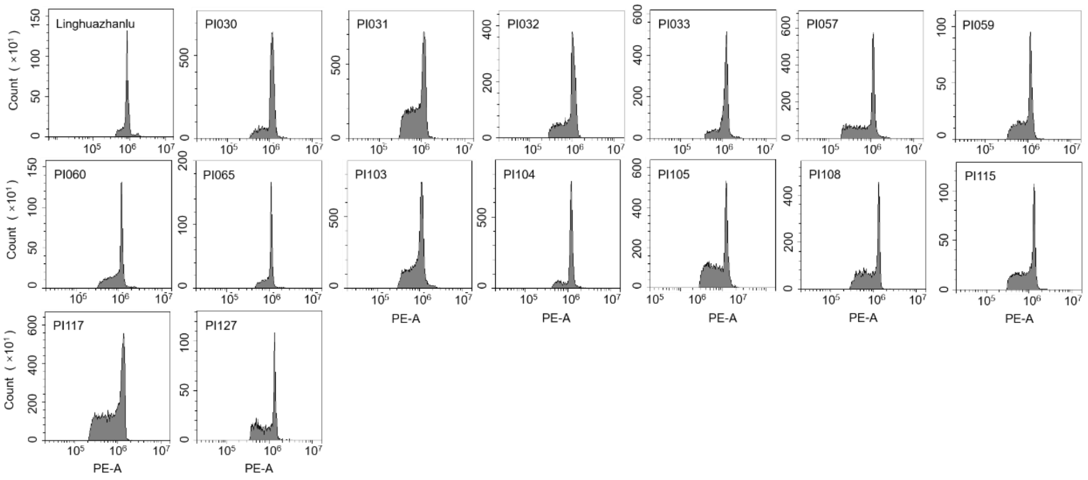

Supplementary Figure 3. Flow cytometric analysis of 15 triploid *P. lactiflora* varieties using diploid cultivars 'Linghuazhanlu' as a reference.

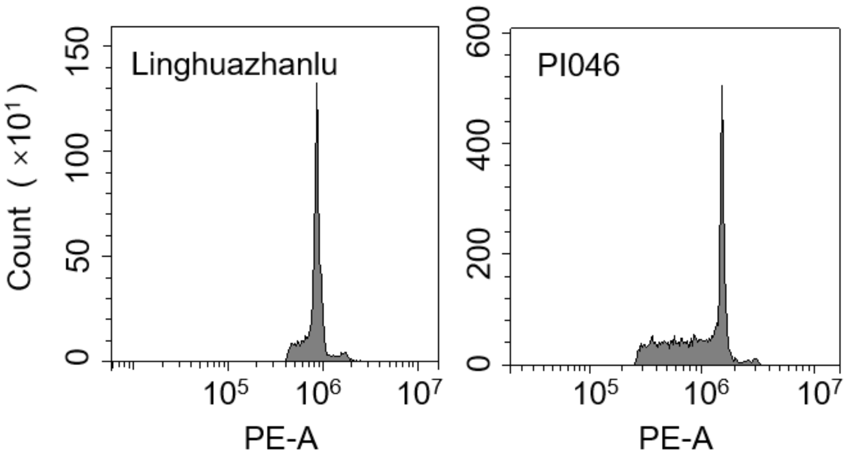

Supplementary Figure 4. Flow cytometric analysis of 1 tetraploid *P. lactiflora* varieties using diploid cultivar 'Linghuazhanlu' as a reference.

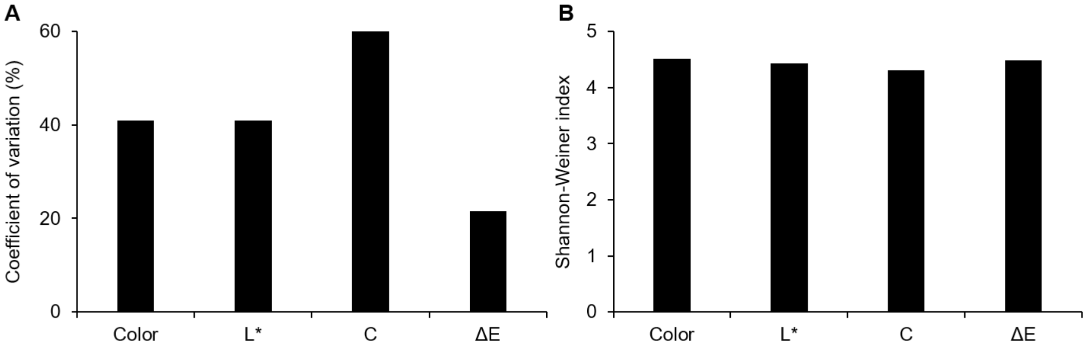

Supplementary Figure 5. Outer-petal color parameter variations in 99 *P. lactiflora* accessions. (A) Variations of color-related traits. (B) Diversity of color-related traits.

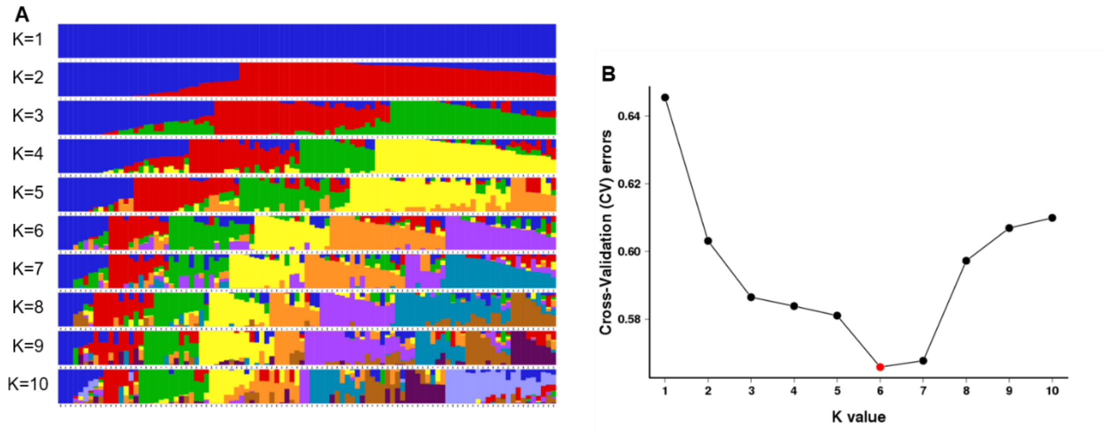

Supplementary Figure 6. Population structure analysis of *P. lactiflora* population. (A) Population structure analysis of 99 *P. lactiflora* accessions. Each vertical column with different color represents an accession. (B) The number of ancestry kinships (K) set to 1~10.

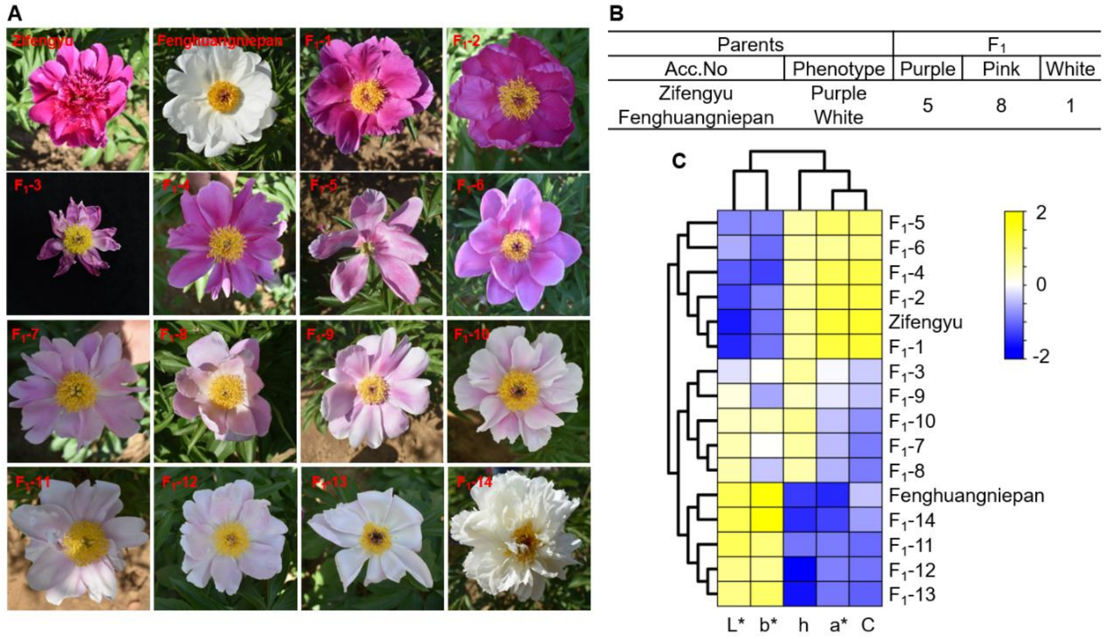

Supplementary Figure 7. Flower phenotype (A and B) and color parameters (C) of the two *P. lactiflora* parental lines and F_1 hybrid population.
